# Thy1.2 YFP-16 Transgenic Mouse Labels a Subset of Large-Diameter Sensory Neurons that Lack TRPV1 Expression

**DOI:** 10.1371/journal.pone.0119538

**Published:** 2015-03-06

**Authors:** Thomas E. Taylor-Clark, Kevin Y. Wu, Julie-Ann Thompson, Kiseok Yang, Parmvir K. Bahia, Joanne M. Ajmo

**Affiliations:** Department of Molecular Pharmacology and Physiology, Morsani College of Medicine, University of South Florida, Tampa, Florida, United States of America; Boston Children’s Hospital and Harvard Medical School, UNITED STATES

## Abstract

The Thy1.2 YFP-16 mouse expresses yellow fluorescent protein (YFP) in specific subsets of peripheral and central neurons. The original characterization of this model suggested that YFP was expressed in all sensory neurons, and this model has been subsequently used to study sensory nerve structure and function. Here, we have characterized the expression of YFP in the sensory ganglia (DRG, trigeminal and vagal) of the Thy1.2 YFP-16 mouse, using biochemical, functional and anatomical analyses. Despite previous reports, we found that YFP was only expressed in approximately half of DRG and trigeminal neurons and less than 10% of vagal neurons. YFP-expression was only found in medium and large-diameter neurons that expressed neurofilament but not TRPV1. YFP-expressing neurons failed to respond to selective agonists for TRPV1, P2X_2/3_ and TRPM8 channels in Ca^2+^ imaging assays. Confocal analysis of glabrous skin, hairy skin of the back and ear and skeletal muscle indicated that YFP was expressed in some peripheral terminals with structures consistent with their presumed non-nociceptive nature. In summary, the Thy1.2 YFP-16 mouse expresses robust YFP expression in only a subset of sensory neurons. But this mouse model is not suitable for the study of nociceptive nerves or the function of such nerves in pain and neuropathies.

## Introduction

Transgenic mice selectively expressing fluorescent proteins in neurons have been developed to study many aspects of neuronal structure and connectivity. These transgenic models have the advantage over other methods of visualization (e.g. immunohistochemistry of neuronal specific proteins), given that they do not require biochemical processing and the fluorescent proteins produce robust fluorescent signals. Furthermore, nerve subsets can be labeled with fluorescent proteins either randomly by random insertion of neuronal promoters [1], or specifically via Cre recombinase systems [2,3].

Sensory nerves are critical for the detection of external and internal environments in multicellular organisms. Peripheral terminals of sensory nerve detect stimuli and conduct this information to synapses within the CNS, in order to elicit reflexes, sensations, behaviors and emotions. To further understand these systems, a series of transgenic animals expressing fluorescent proteins in random subsets [1] has been used repeatedly to study sensory nerve terminal structure, sensory nerve connections in the spinal cord, sensory nerve development and axonal loss in neuropathies [4,5,6,7,8,9]. However, sensory nerves are heterogeneous with respect to protein expression and function and it is not clear the extent to which fluorescent proteins in these transgenic mice label select sensory subtypes involved in distinct reflex and behavioral pathways [1].

Sensory nerves are generally divided into 2 main subsets: nerves that respond to noxious stimuli such as noxious heat and acid (often termed nociceptors) and nerves that respond to non-noxious stimuli such as light touch (often termed low-threshold mechanosensors or non-nociceptors) [10,11]. Nociceptors typically have small-diameter cell bodies [12]; unmyelinated or weakly myelinated axons; and almost exclusively express the canonical nociceptive ion channel TRPV1, which is selectively activated by capsaicin, the pungent ingredient of chili peppers [13]. Non-nociceptive neurons typically have large-diameter cell bodies, myelinated axons, express medium and heavy neurofilament but not TRPV1. Sensory neurons residing in the dorsal root ganglia (DRG) innervate the skin (below the neck), skeletal muscle and viscera in the thorax and abdomen. Trigeminal sensory neurons innervate the cranial skin and viscera and vagal neurons innervate the viscera in the thorax and abdomen.

Here, we have characterized the expression of yellow fluorescent protein (YFP) in sensory nerves in the Thy1.2 YFP-16 mouse [1], a commonly used transgenic model [6,7,8,9]. YFP expression was restricted to large-diameter neurons that expressed neurofilament 200 (NF200) but did not express TRPV1 or respond to capsaicin (TRPV1), menthol (TRPM8) or α,β methylene ATP (P2X_2/3_), suggesting that only non-nociceptive neurons were labeled. Numerous YFP expressing neurons were found in the DRG and trigeminal ganglia, but only a few were found in the vagal ganglia. YFP was expressed in terminals innervating hair follicle terminals in hairy skin, in terminals innervating Meissner corpuscles in glabrous skin and in terminals of skeletal muscle spindles.

## Results

**Fig 1:**
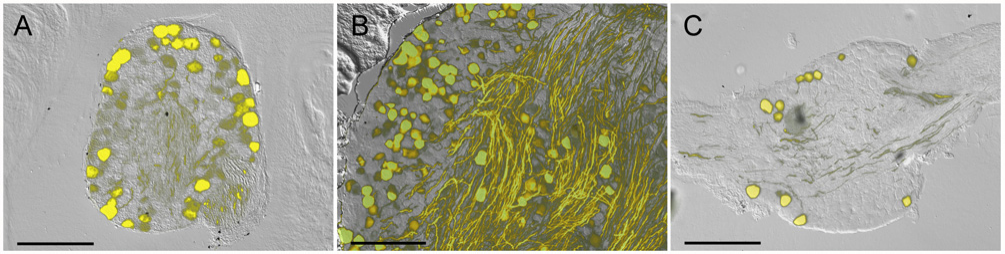
Fluorescence imaging of YFP in sensory ganglia of Thy1.2 YFP-16 mice. *A*, DRG. *B*, Trigeminal ganglion. *C*, vagal ganglion. YFP expression is shown in yellow overlaid upon bright field image. Scale bar represents 200 μm.

We began by determining the expression of YFP in sensory ganglia in the Thy1.2 YFP-16 mice. Serial frozen sections from DRG, trigeminal and vagal ganglia showed that YFP was present in neuronal cell bodies and axons but not in other cell types. Contrary to previous studies [1] we found that not all sensory neurons expressed YFP. Approximately half of DRG and trigeminal neurons and less than 10% of vagal neurons expressed YFP (Fig. 1). YFP-expressing axons were visible in all ganglia, but were particularly prevalent in the trigeminal. Some of these trigeminal axons may be motor fibers [1], derived from cranial motor cell bodies that do not reside in the trigeminal ganglia.

**Fig 2:**
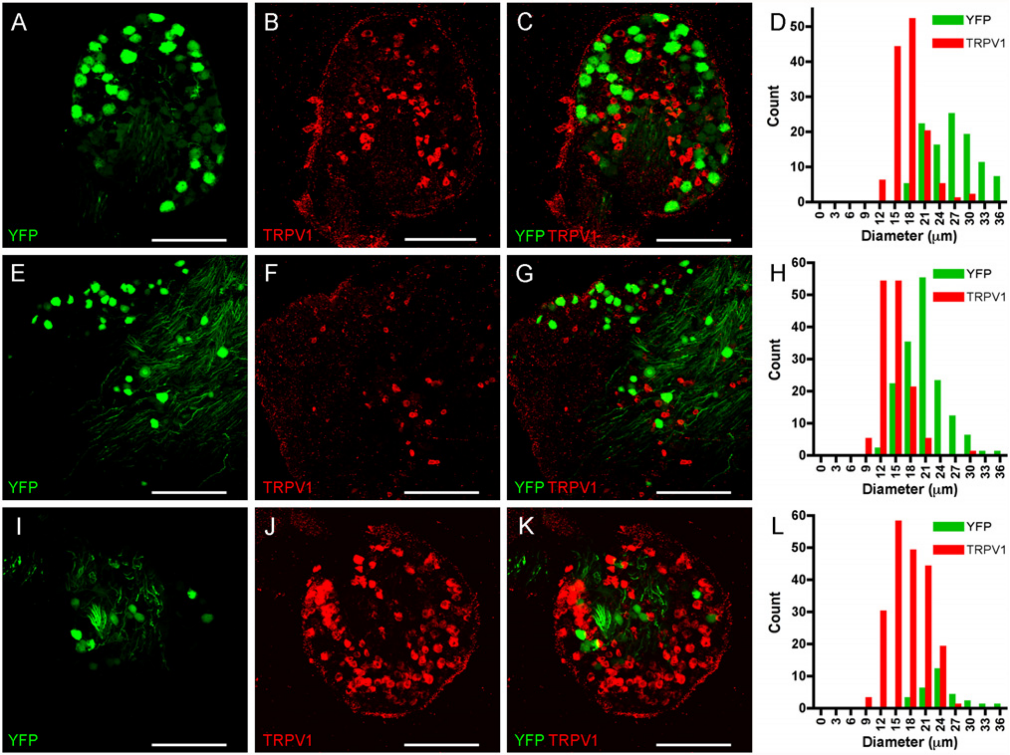
YFP and TRPV1 expression do not co-localize in sensory neurons. *A-D*, DRG. *E-H*, Trigeminal ganglion. *I-L*, vagal ganglion. YFP expression is shown in green (*A*, *E*, *I*), TRPV1 expression is detected immunohistochemically and shown in red (*B*, *F*, *J*), overlay showing distinct expression patterns (*C*, *G*, *K*). Scale bar represents 200 μm. *D*, *H and L*, histogram of neuronal diameters.

We hypothesized that the selective expression of YFP in subpopulations of sensory nerves was correlated to sensory nerve phenotype. In general, nociceptive nerves have small diameter cell bodies and respond to capsaicin (due to their selective expression of TRPV1), but do not express significant levels of heavy chain neurofilament (NF200). Non-nociceptive nerves have large diameter cell bodies, have significant expression of NF200 but do not express TRPV1. First, we performed confocal microscopy of sensory ganglia from Thy1.2 YFP-16 mice and investigated the expression of TRPV1 using immunohistochemistry. As before YFP was expressed in many DRG and trigeminal neurons, but only a few vagal neurons. Anti-TRPV1 antibodies labeled a completely distinct population of sensory neurons in all sensory ganglia (Fig. 2), which was absent in slides processed without primary antibody against TRPV1 (data not shown). We did not detect a single neuron that expressed both YFP and TRPV1 in the sensory ganglia. In DRG we counted 105 YFP-expressing neurons and 130 TRPV1-expressing neurons (in 4 ganglia from 3 animals, Fig. 2A-D). Consistent with previously published correlations of nociceptive vs. non-nociceptive neuronal diameters, the YFP-expressing neurons were significantly larger than the TRPV1-expressing neurons (26.6 ± 0.47 μm vs. 17.7 ± 0.27 μm, p<0.0001). In trigeminal ganglia we counted 156 YFP-expressing neurons and 140 TRPV1-expressing neurons (in 3 ganglia from 3 animals, Fig. 2E-H). Again, the YFP-expressing neurons were significantly larger than the TRPV1-expressing neurons (20.6 ± 0.31 μm vs. 14.4 ± 0.24 μm, p<0.0001). Finally, in vagal ganglia we counted 28 YFP-expressing neurons and 204 TRPV1-expressing neurons (in 4 ganglia from 3 animals, Fig. 2I-L). As before, the YFP-expressing neurons were significantly larger than the TRPV1-expressing neurons (24.0 ± 0.7 μm vs. 17.5 ± 0.26 μm, p<0.0001).

**Fig 3:**
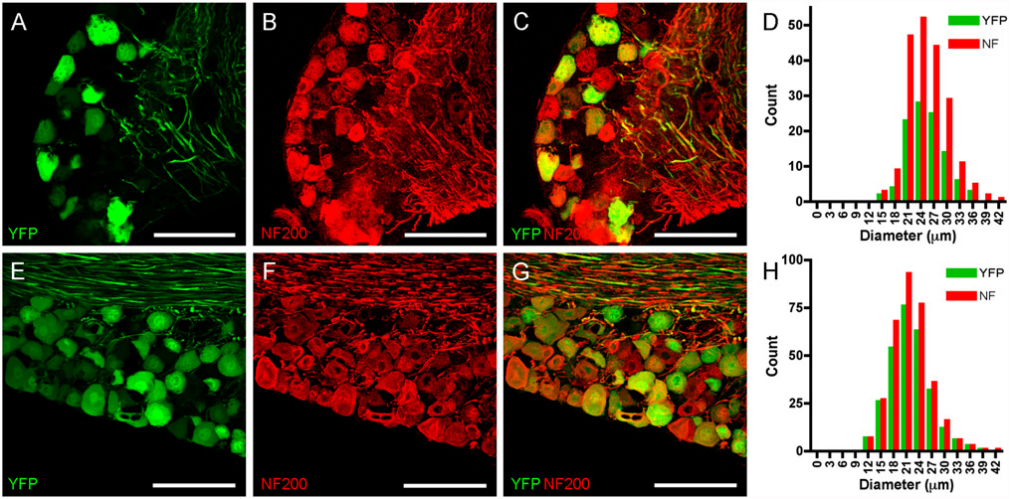
YFP co-localizes with NF200 in sensory neurons. *A-D*, DRG. *E-H*, Trigeminal ganglion. YFP expression is shown in green (*A and E*), NF200 expression is detected immunohistochemically and shown in red (*B and F*), overlay showing almost identical expression patterns (*C and G*). Scale bar represents 100 μm. *D and L*, histogram of neuronal diameters.

We also investigated the expression of the non-nociceptive marker NF200 in confocal microscopy of DRG and trigeminal ganglia from Thy1.2 YFP-16 mice and compared it with YFP expression. Anti-NF200 antibodies labeled many large diameter sensory neurons in both sensory ganglia (Fig. 3), which was again absent in slides processed without primary antibody against NF200 (data not shown). Almost all YFP-expressing neurons were also positive for NF200. In DRG we counted 105 YFP-expressing neurons, and all but one of these neurons also expressed NF200 (in 7 ganglia from 3 animals, Fig. 3A-D). In total, we counted 202 NF200-expressing DRG neurons of which 51% expressed YFP. YFP- and NF200-expressing DRG neurons were not significantly different in diameter compared to the other NF200-expressing DRG neurons (25.3 ± 0.41 μm vs. 25.3 ± 0.46 μm, p>0.2). Similarly, in trigeminal ganglia we counted 280 YFP-expressing neurons, and all but 6 of these neurons also expressed NF200 (in 3 ganglia from 3 animals, Fig. 3E-H). In total, we counted 334 NF200-expressing trigeminal neurons of which 82% expressed YFP. YFP- and NF200-expressing DRG neurons were not significantly different in diameter compared to the other NF200-expressing DRG neurons (21.8 ± 0.28 μm vs. 21.9 ± 0.46 μm, p>0.2). In summary, the immunohistochemical analysis indicates that YFP expression is restricted to a subset of large-diameter NF200-expressing neurons that do not express TRPV1.

We next studied the correlation of YFP expression in sensory ganglia with functional responses to sensory nerve stimuli. Using Fura 2AM Ca^2+^ imaging in dissociated neurons we tested the sensitivity of these sensory neurons to the selective TRPV1 agonist capsaicin (1 μM) and the selective P2X_2/3_ agonist α,β methylene ATP (10 μM). Both these channels are Ca^2+^ permeable when activated. Capsaicin identifies nociceptive neurons [13], whereas α,β methylene ATP identifies a subset of non-peptidergic sensory neurons [14,15] which, in the case of the vagal ganglia, are derived embryologically from the cranial placodes [16].

**Fig 4:**
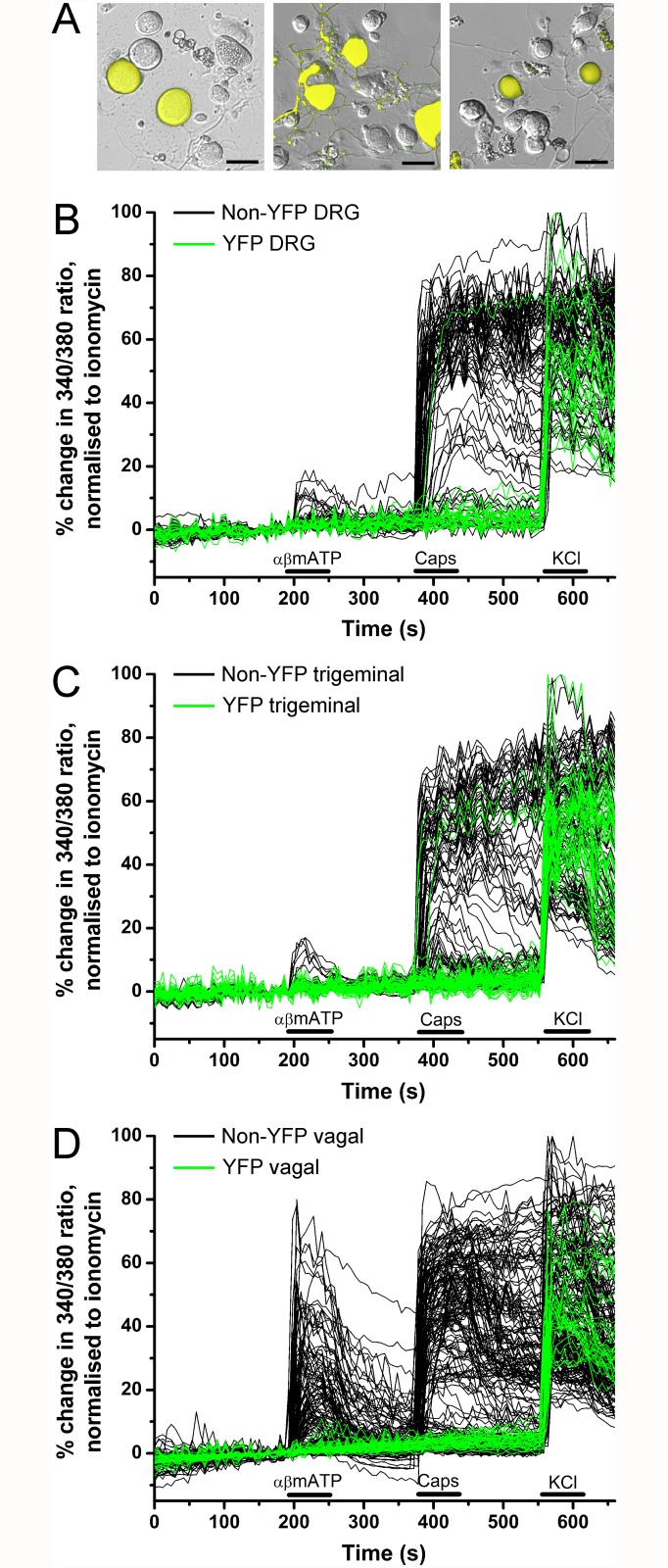
YFP-expressing sensory neurons are insensitive to capsaicin and α,β methylene ATP. *A*, representative fluorescent images of YFP expression (yellow) in dissociated neurons overlaid upon bright field image: DRG (*left*), trigeminal (*middle*) and vagal (*right*). Scale bar represents 35 μm. *B-D*, Ca^2+^ responses of individual dissociated DRG neurons (*A*), trigeminal ganglia neurons (*B*) and vagal ganglia neurons (*C*) in response to α,β methylene ATP (10 μM), capsaicin (Caps, 1 μM,) and KCl (75 mM). Green lines for YFP neurons, black lines for non-YFP neurons. Blocked lines depict the duration of drug application.

**Fig 5:**
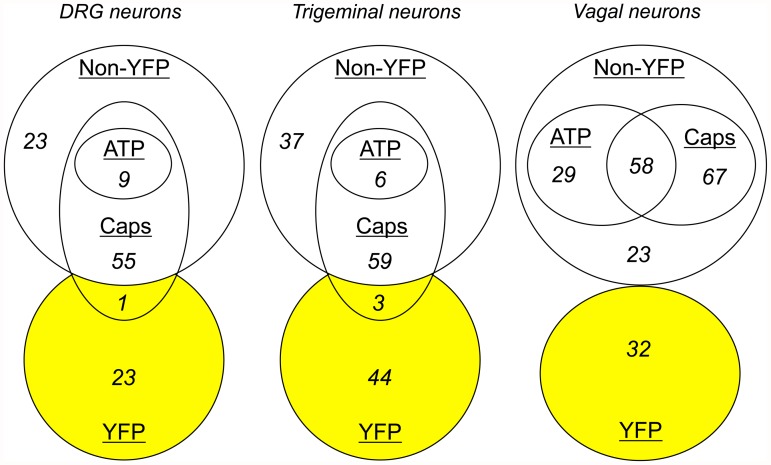
Euler diagrams comparing sensory nerve populations with YFP expression. Populations ascribed by sensitivity to α,β methylene ATP (10 μM) and capsaicin (Caps, 1 μM,).

In experiments with dissociated DRG neurons (from 9 ganglia from 3 animals), we recorded from 24 YFP-expressing neurons and 87 non-fluorescent neurons (Figs. 4A and 5). Only 1 out of 24 YFP-expressing neurons responded to capsaicin, and none responded to α,β methylene ATP. The majority of non-fluorescent neurons responded to capsaicin (64 out of 87), with only 9 of these also responding to α,β methylene ATP. Consistent with our confocal imaging of the DRG slices, the dissociated YFP-expressing DRG neurons were larger than both the entire non-fluorescent DRG population (34.4 ± 1.1 μm vs. 23.9 ± 0.53 μm, p<0.0001) and the subpopulation of non-fluorescent DRG that failed to respond to either capsaicin or α,β methylene ATP (vs. 25.5 ± 1.2 μm, p<0.0001).

In experiments with dissociated trigeminal neurons (from 6 ganglia from 3 animals), we recorded from 47 YFP-expressing neurons and 102 non-fluorescent neurons (Figs. 4B and 5). Only 3 out of 47 YFP-expressing neurons responded to capsaicin, and none responded to α,β methylene ATP. Again, the majority of non-fluorescent neurons responded to capsaicin (65 out of 102), with only 6 of these also responding to α,β methylene ATP. The dissociated YFP-expressing trigeminal neurons were larger than both the entire non-fluorescent trigeminal population (25.4 ± 0.77 μm vs. 19.1 ± 0.26 μm, p<0.0001) and the subpopulation of non-fluorescent trigeminal that failed to respond to either capsaicin or α,β methylene ATP (vs. 19.5 ± 0.47 μm, p<0.0001).

With vagal neurons (from 8 ganglia from 4 animals), we recorded responses to capsaicin and α,β methylene ATP from 32 YFP-expressing neurons and 196 non-fluorescent neurons (Figs. 4C and 5). None of the YFP-expressing neurons responded to either capsaicin or α,β methylene ATP. The majority of non-fluorescent neurons responded to capsaicin (125 out of 196). Neuronal activation by α,β methylene ATP was far more prevalent in vagal non-fluorescent neurons compared to DRG or trigeminal neurons with significant responses in 46% of capsaicin-sensitive neurons and 41% of capsaicin-insensitive neurons. The dissociated YFP-expressing vagal neurons were larger than both the entire non-fluorescent vagal population (28.2 ± 0.81 μm vs. 20.7 ± 0.34 μm, p<0.0001) and the subpopulation of non-fluorescent vagal that failed to respond to either capsaicin or α,β methylene ATP (vs. 21.4 ± 0.60 μm, p<0.0001). We also determined the sensitivity of vagal neurons to 100μM menthol, the selective agonist for the cold-sensitive receptor TRPM8. Menthol activated 0 out of 33 YFP-expressing vagal neurons. However, menthol activated 57 out of 137 non-fluorescent vagal neurons, 48 of which were also capsaicin-sensitive (data not shown).

**Fig 6:**
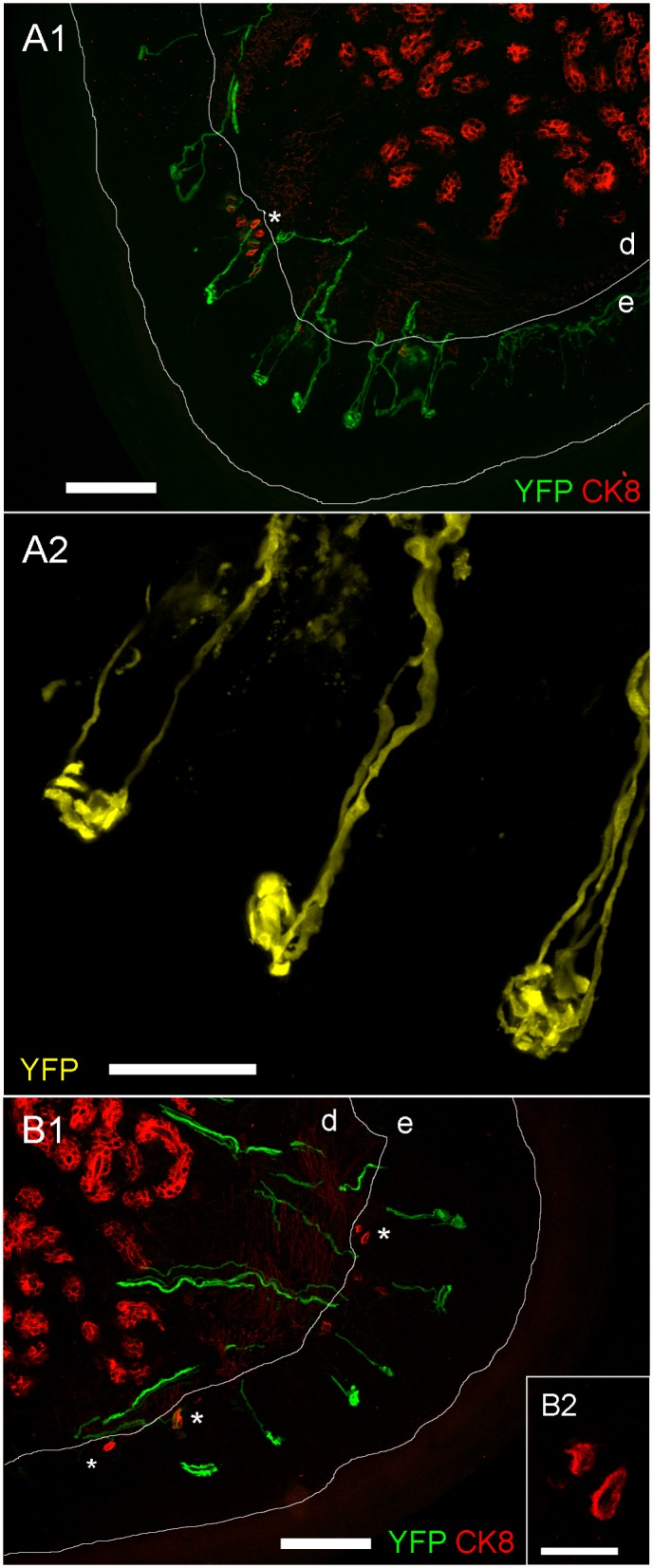
Confocal imaging of YFP expression in glabrous skin nerve terminals of the hind paw. *A1 and B1*, multiple YFP-expressing sensory terminals within the epidermis (e). CK8-positive Merkel cells (denoted by *) are located at the basal epidermal layer. Other CK8-positive cells are found within the dermis (d). YFP is shown in green and CK8 is shown in red, scale bar represents 100 μm. *A2*, fine structure of YFP-expressing sensory terminals within the epidermis. YFP is shown in yellow, scale bar represents 25 μm. *B2*, fine structure of CK8-positive Merkel cells, scale bar represents 20 μm.

**Fig 7:**
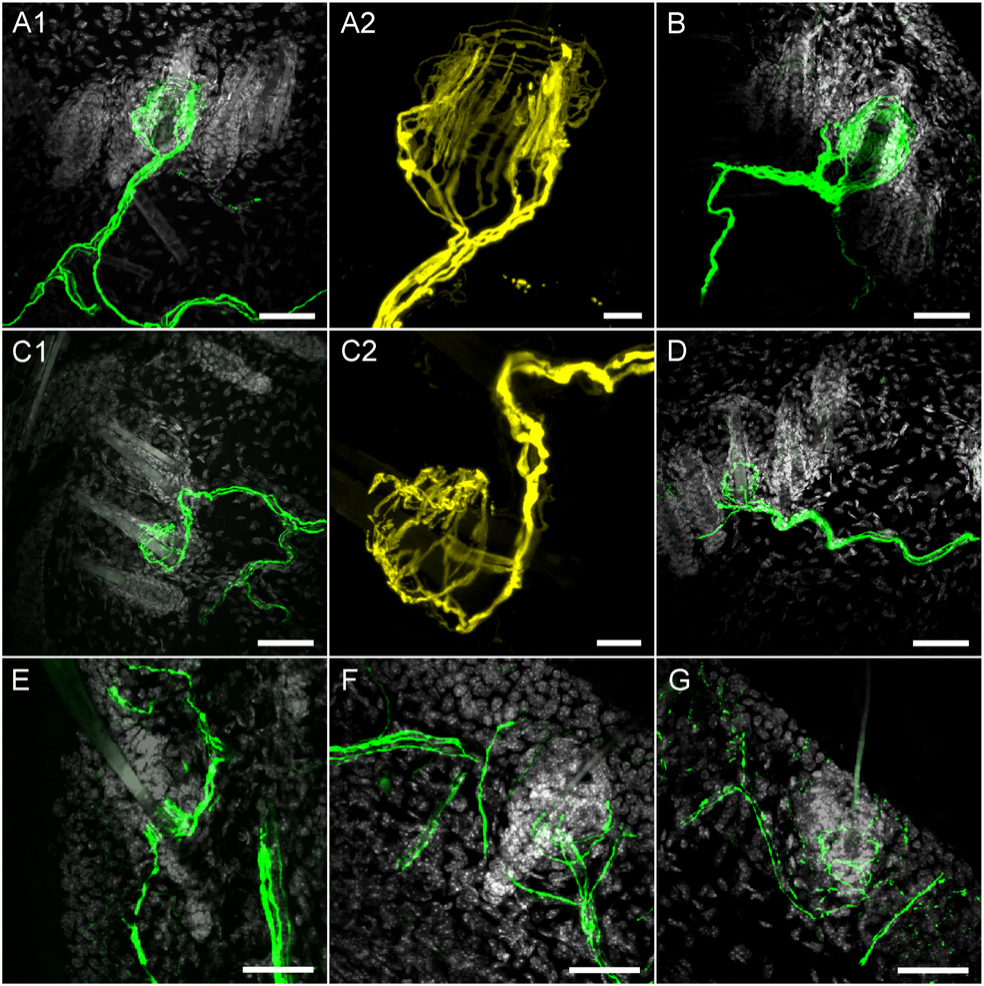
Confocal imaging of YFP expression in hairy skin nerve terminals. *A1*, *A2 and B*, YFP-expressing longitudinal lanceolate endings innervating hair on dorsal skin. *C1*, *C2 and D*, YFP-expressing circumferential lanceolate endings innervating hair on dorsal skin. *E*, YFP-expressing longitudinal lanceolate ending innervating hair on ear skin. *F and G*, YFP-expressing circumferential lanceolate endings innervating hair on ear skin. *A1*, *B*, *C1*, *D-G*: YFP is shown in green and DAPI is shown in greyscale, scale bar represents 40 μm. *A2 and C2*: YFP is shown in yellow, scale bar represents 10 μm.

**Fig 8:**
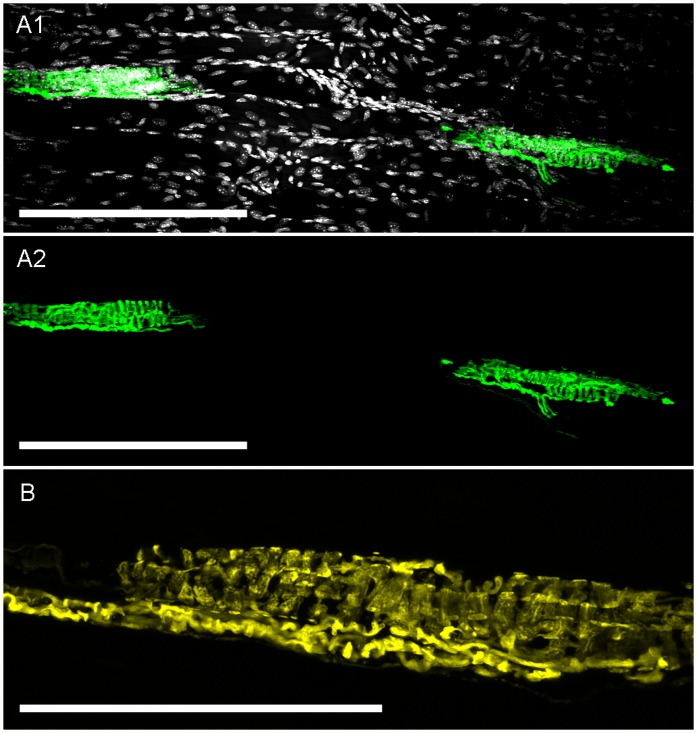
Confocal imaging of YFP expression in skeletal muscle spindles. *A1*, YFP-expressing muscle spindles in deep tissue of tibialis anterior skeletal muscle. YFP is shown in green and DAPI is shown in greyscale, scale bar represents 200 μm. *A2*, as *A1* but without DAPI staining for clarity. *B*, fine structure of another YFP-expressing muscle spindle. YFP is shown in yellow, scale bar represents 100 μm.

Lastly, we investigated the structure of peripheral YFP-expressing sensory nerve terminals. Given that YFP-expression was prevalent in DRG and trigeminal neurons, but not in vagal neurons, we concentrated on structures known to be innervated by these types of sensory receptors: cutaneous terminals in glabrous skin of the paw and the hairy skin of the dorsal skin and muscle spindles of skeletal muscle (all innervated by DRG); and the hairy skin of the ear (innervated by the trigeminal). Confocal imaging of serial frozen sections of these tissues (from 6 animals) showed YFP expression in distinct nerve terminal structures (Figs. 6–8).

Numerous YFP-expressing terminals were found in glabrous skin of the hindpaw (Fig. 6A1, 6B1). The greatest density was found at the fingertips and on the central footpad dome, but similar terminals were found across the full glabrous skin surface. The nerves uniformly terminated in endings in the middle of the epidermal layer, and these terminals were composed of complex corkscrew-like structures approximately 10 to 30 μm in diameter (Fig. 6A2). Cytokeratin 8 (CK8) is a marker for Merkel cells [17,18]. As expected we found CK8-positive Merkel cells in small clusters at the basement layer of the epidermis. These small mechanotransductive cells (approximately 10 μm in diameter) (Fig. 6B2) were not innervated by YFP-expressing terminals. We also found significant CK8-positive staining deep in the dermis, which is consistent with the expression of CK8 in other cell types other than Merkel cells. These dermal cells were also not innervated by YFP-expressing terminals. We found identical YFP-expressing structures in the glabrous skin of the forepaw (data not shown).

We found YFP-expressing terminals innervating the roots of some hair follicles in both dorsal skin and ear skin. It was clear that the hair follicles were innervated by two distinct populations of YFP-expressing nerves: terminals with longitudinal lanceolate endings (Fork-like structures; Fig. 7A, 7B and 7E) and terminals with circumferential lanceolate endings (Fig. 7C, 7D, 7F and 7G). In the dorsal skin we counted 1356 hairs (from 3 animals). Out of this total only 20 hairs (diameter 19.04 ± 0.84 μm) were innervated by YFP-expressing longitudinal lanceolate endings, and only 14 hairs (diameter 12.80 ± 0.78 μm, p<0.01) were innervated by YFP-expressing circumferential lanceolate endings. The size and structure of these hairs indicate that YFP-expressing longitudinal lanceolate endings innervated tylotrich (guard) hairs and that non-tylotrich hairs (e.g. awl/auchene) were innervated by YFP-expressing circumferential lanceolate endings. Both types of lanceolate endings had well-defined structures that completely encircled the hair follicle (Fig. 7A-D). These structures were approximately 25–55 μm in diameter, with longitudinal lanceolate endings being in general larger than circumferential lanceolate endings. Due to the rarity of YFP-expressing endings and the orientation and thickness of the sections it was not possible to determine if a single nerve innervated only a single hair, or a diffuse network of hairs.

In the ear skin, YFP-expressing terminal structures were less well-defined, particularly the circumferential lanceolate endings which often innervated only one side of the follicle (Fig. 7F and 7G). The diameter of the hair follicles innervated by YFP-expressing endings in the ear skin was smaller than in dorsal skin (7.51 ± 0.79 μm vs. 16.00 ± 0.76 μm, p<0.001). Again, less than 3% of hairs were innervated by YFP-expressing endings.

Lastly, we found YFP expression in muscle spindles throughout the tibialis anterior muscle (Fig. 8). These muscle spindles (approximately 150μm in length) were innervated by one or more YFP-expressing axons. No other YFP-expressing sensory receptors were found in the skeletal muscle, including the tendons (data not shown).

## Discussion

The Thy1.2 YFP-16 mouse model has been used in multiple studies of sensory nerve function [6,7,8,9]. The model was created by incorporation of YFP under the control of the neuronal Thy1.2 promoter [1]. Although the initial incorporation event was random, progeny from the mouse displayed consistent neuronal YFP expression. Originally this mouse model was reported to express YFP in all sensory nerves and somatic motor nerves but rarely in postganglionic autonomic nerves [1], thus making it a useful tool to study sensory nerves. Here, we have characterized the expression of YFP in sensory neurons from the DRG, trigeminal ganglia and vagal ganglia and we report that only a subset of these sensory nerves express YFP, based upon biochemical, functional and anatomical analysis.

YFP expression was observed in approximately 40–50% of DRG and trigeminal neurons based upon fluorescent and confocal imaging of frozen sections. In vagal ganglia, only a small number of neurons expressed YFP. Analysis of TRPV1 expression (nociceptor marker [13]), NF200 expression (non-nociceptive marker) and the size of the neuronal cell bodies [12] suggest that YFP was solely expressed in non-nociceptive sensory neurons. A significant population of large, NF200-positive sensory neurons failed to express YFP, suggesting that YFP was only expressed in a subset of non-nociceptive neurons.

Consistent with the fixed tissue studies, functional studies of dissociated sensory neurons suggest that YFP was indeed expressed in a subset of non-nociceptive neurons. Throughout all sensory ganglia we recorded from 135 YFP-expressing neurons and only 4 responded to capsaicin (~3%), whereas 343 out of 522 non-fluorescent neurons were capsaicin-sensitive (~66%). Supporting this data, the YFP-expressing neurons were significantly larger than non-fluorescent neurons. It is possible that the rare capsaicin-sensitivity of YFP-expressing neurons was due to culture-induced *de novo* expression of TRPV1 in neurons that would normally be TRPV1-negative in vivo. Such minor neuroplasticity has been shown previously to be sensitive to neurotrophins and culture conditions [19]. It should be noted that YFP expression may also be sensitive to culture conditions. Specific YFP expression in dissociated neurons and ganglionic slices is difficult to compare rigorously because only those neurons in the field of view are functionally assayed and selection of the field of view was biased by YFP expression (but not functional responses, which were reported for every neuron assayed).

We also studied the sensitivity of dissociated neurons to α,β methylene ATP, the selective agonist of heteromeric P2X_2/3_ channels. P2X_2_ subunits are only expressed in a subset of non-peptidergic neurons [14,15]. P2X_2_ subunits combine with P2X_3_ in these neurons to form the heteromeric P2X_2/3_ channel. In the vagal ganglia, which comprises of two ganglia (nodose and jugular) derived from different embryological sources (epibranchial placodes and neural crest, respectively), P2X_2_ expression correlates with the nodose/placodal phenotype [16,20]. Given that many vagal neurons are nodose/placodal in origin, we were not surprised to find that a large number of vagal dissociated neurons (~44% in total) responded to α,β methylene ATP, unlike the neural crest-derived DRG (~10%) and trigeminal (~6%). Despite the high percentage of α,β methylene ATP-sensitivity in vagal capsaicin-insensitive neurons, YFP-expressing vagal neurons were insensitive to this P2X_2/3_ agonist. This data suggests that YFP is only expressed in neural crest-derived non-nociceptive neurons and this may explain why YFP expression was only observed in a small number of vagal neurons (which is a combination of placodal and neural crest neurons) compared to DRG and trigeminal (which are neural crest in origin) (see Fig. 1).

Peripheral terminals of sensory nerves display different anatomical structures of various complexity and size that correlates with sensory function [11,21,22,23]. Non-nociceptive sensory nerves innervate structures throughout the body including the skin and skeletal muscle. In the skin, some sensory nerves serve as low-threshold mechanoreceptors (LTMRs) that convey mechanical information regarding brush, stretch, vibration, hair deflection and pressure. These diverse sensations are known to be triggered by activation of different classes of LTMRs including myelinated fast Aβ fibers innervating Ruffini corpuscles, Pacinian corpuscles, Meissner corpuscles, Merkel cells and thick Guard hair follicles; myelinated fast Aδ fibers innervating lighter hair follicles; and unmyelinated slow C fibers innervating lighter hair follicles [11,23]. In skeletal muscle, myelinated sensory nerves innervate both the intrafusal fibers of muscle spindles (which senses muscle stretch) and the Golgi tendon organ (which senses muscle tension) [21].

In the glabrous skin of Thy1.2 YFP-16 mice, we found that only one type of ending expressed YFP: a characteristic and complex corkscrew-like ending innervating half-way through the epidermis, which were approximately 10–30 μm in diameter. The location in the epidermis, size and shape of these numerous YFP-expressing endings suggest that they are Meissner corpuscles [24,25]. CK8 (also known as Troma1) is a marker for Merkel cells [17,18] and, as expected, these were found individually or in small clusters throughout the basal layer of the epidermis of glabrous skin [24,26]. Merkel cells were not innervated by the YFP-expressing intraepidermal endings. In addition, we suggest that the YFP-expressing endings are not Ruffini corpuscles or Pacinian corpuscles, which are much larger and are located in the dermis [24]. Sensory nerves innervating Meissner corpuscles in glabrous skin are thought to be rapidly-adapting LTMRs that sense dynamic skin deformation [11,23].

We failed to observe YFP-expressing corkscrew sensory endings in hairy skin of Thy1.2 YFP-16 mice, consistent with the fact that Meissner corpuscles are not found in hairy skin [11,23]. Nevertheless we observed two distinct subtypes of YFP-expressing endings innervating a small percentage of hair follicles in dorsal skin. Firstly, we found YFP-expressing longitudinal lanceolate endings innervating the follicle of large guard hairs. These structures invariably surrounded the entire follicle and they are very similar in structure to longitudinal lanceolate endings ascribed to Aβ fibers innervating rapidly-adapting LTMR that sense hair displacement [7,18]. The second type of YFP-expressing terminal were circumferential lanceolate endings, which also surrounded the entirety of the follicle. The size of these particular follicles suggest that they are non-tylotrich hairs (likely to be awl/auchene hairs). Such endings have been described previously [7,27,28], but their function is presently unknown [11]. Comparisons between the YFP-expressing endings found in dorsal skin and ear skin suggest that the structure of trigeminal LTMR endings is more disorganized than DRG LTMR endings. The reason for this is unknown, but the hairs innervated by YFP-expressing endings on the ear were much thinner than those found on the dorsal skin, consistent with the lack of thick guard hairs on the ear.

In skeletal muscle we found muscle spindles (and their innervating axons) expressed YFP. Muscle spindles are located in intrafusal fibers of skeletal muscle in parallel with the main contractile extrafusal muscle fibers. Muscle spindles detect skeletal muscle stretch and they respond in a rapidly adapting manner [21]. The location, shape and size of the YFP-expressing sensory structures found in the tibialis anterior muscle of Thy1.2 YFP-16 mice are identical to muscle spindle structures identified in other mice strains [21,29,30]. We failed to observe YFP-expressing afferents or structures in the tendons, thus we suggest that the Golgi tendon organ afferents [31] do not express YFP in this mouse model.

In our neuronal studies it was clear that some TRPV1-negative/capsaicin-insensitive/NF200-positive neurons did not express YFP. This is consistent with our nerve terminal studies that showed that some LTMR subtypes were not fluorescent (e.g. fibers innervating Ruffini corpuscles, Pacinian corpuscles, Merkel cells and Golgi tendon organs) and not many hair follicles were innervated by YFP-expressing endings. It is not clear why only a few LTMR subtypes express YFP, given that LTMR share the same characteristic of lack of TRPV1 and P2X_2_ expression.

The original characterization of the expression of Thy1.2 YFP-16 mice was part of a massive study involving 25 individual Thy1.2 transgenic models [1]. Expression of fluorescent proteins in sensory neurons was reported for only the DRG. The authors reported “all” DRG neurons in the Thy1.2 YFP-16 mice were fluorescent, but no images were published for this data. Nevertheless, images of fluorescent protein expression in the DRG was reported for the YFP-G, GFP-I, CFP-4 and GFP-O models. Although these models had different levels of expression, it is clear from the images that large neurons were preferentially fluorescent. This is consistent with our data that YFP is expressed preferentially in TRPV1-negative/capsaicin-insensitive/NF200-positive neurons.

In summary, we have characterized the sensory nerve expression of YFP in the Thy1.2 YFP-16 mouse. Contrary to previous reports, we have found that only certain TRPV1-negative neurons express YFP. YFP expression was robust and allowed for detailed analysis of specific peripheral terminal structures. However, this mouse model is not suitable for the study of nociceptive nerves or the function of such nerves in pain and neuropathies.

## Materials and Methods

### Animals

All experiments were conducted with male 5–7 week old Thy1.2 YFP-16 mouse. Breeding pairs of the B6.Cg-Tg(Thy1-YFP)16Jrs/J strain were purchased from Jackson Laboratory (Maine) and their progeny were used for all studies in accordance with and approved by the University of South Florida Institutional Animal Care and Use Committee. Prior to tissue harvest, mice were killed by CO_2_ asphyxiation followed by exsanguination. DRG, trigeminal ganglia and vagal ganglia were immediately isolated prior to fixation with 4% paraformaldehyde (in phosphate-buffered saline (PBS), 2 hours, 4°C) or enzymatic dissociation (see below). In addition, the skin from the forepaw and hindpaw, 5 mm square clips of mice ears, 8 mm square sections of dorsal skin and the tibialis anterior muscle were dissected and fixed in 4% paraformaldehyde (in PBS, 2 hours, 4°C).

### Tissue processing

Following fixation, tissue was cryoprotected overnight in 18% sucrose (in PBS, 4°C) then immobilized in Tissue Freezing Medium (Triangle Biomedical Services, North Carolina). Ganglia were sectioned in 20 μm slices, skin samples were sectioned in 40–80 μm slices, skeletal muscle were sectioned in 40 μm slices. For immunohistochemical detection of specific proteins, sections were blocked with donkey serum (10% in PBS with 1% bovine serum albumin (BSA), 0.5% Tween 20, 2 h) and incubated overnight (4°C) with appropriate primary. The primary antibody was omitted in 1 slide in 5 as a negative control. Sections were rinsed with PBS containing 0.3% Triton X and 1% BSA and incubated with appropriate secondary antibody for 2 hours at room temperature. TRPV1 was detected with a goat polyclonal antibody to TRPV1 (P-19 (sc-12498), Santa Cruz Biotechnology, Inc., Santa Cruz, CA; 1:150) and Alexa Fluor 594 rabbit anti-goat IgG (Molecular Probes, Inc., Eugene, OR, USA; 1:300). NF200 was detected with a rabbit polyclonal antibody to NF200 (AB1989, EMD Millipore Corporation, Temecula, CA; 1:1000) and Cy5 goat anti-rabbit IgG (Molecular Probes, Inc., Eugene, OR, USA; 1:500). CK8 was detected with a rat monoclonal antibody to CK8 (also known as Troma1) (Developmental Studies Hybridoma Bank (University of Iowa); 1:100) and Alexa Fluor 594 rabbit anti-rat IgG (Molecular Probes, Inc., Eugene, OR, USA; 1:500). Ganglionic sections were coverslipped with VECTASHIELD HardSet Mounting Medium (Vector laboratories, CA, USA). Skin and skeletal muscle sections were coverslipped with VECTASHIELD HardSet Mounting Medium with DAPI.

### Fluorescent YFP imaging

YFP expression was determined using 470 nm excitation (40 nm range) and 525 nm emission (50 nm range) and compared with bright field differential interference contrast measured by digital microscopy (CoolSnap HQ2; Photometrics, Surrey, BC, Canada) with a 10X objective and analyzed by Nikon Elements (Nikon, NY, USA).

### Confocal imaging

Sections were viewed using an Olympus FV1000 laser scanning microscope and Olympus Fluoview 3.0 software with 10X, 20X and 40X objectives where appropriate. DAPI was excited by a 405 nm laser (emission 415–480 nm), YFP was excited by a 515 nm laser (emission 530–620 nm), Alexa Fluor 594 (TRPV1, CK8) was excited by a 543 nm laser (emission 620–720 nm) and Cy5 (NF200) was excited by a 633 nm laser (emission 650–750 nm). Z-stack images were created from individual sections ranging from 0.75 to 3 μm in depth. Neuronal diameters were measured in ImageJ.

### Dissociation of mouse ganglia

Following isolation sensory ganglia were immediately enzymatically dissociated using previously described methods [32]. Isolated neurons were plated onto poly-D-lysine and laminin-coated coverslips, incubated at 37°C in L-15 (supplemented with 10% FBS) and used within 24 hours.

### Fluorescent Ca2+ imaging

Neurons were studied for changes in [Ca^2+^]_i_ with Fura-2AM. Neuron-covered coverslips were incubated (37°C) with Fura-2AM (4 μM, for 30 min) in L-15 media containing 10% FBS. For imaging, the coverslip was placed in a custom-built heated chamber (bath volume of 300 μL) and superfused by gravity at 8 ml/min with HEPES-buffered solution (composition (mM)): 154 NaCl, 4.7 KCl, 1.2 MgCl2, 2.5 CaCl2, 10 HEPES, 5.6 dextrose adjusted to pH 7.4 with NaOH) for 10 minutes before and throughout each experiment. YFP expression was determined using 470 nm excitation (40 nm range) and 525 nm emission (50 nm range) and compared with bright field differential interference contrast measured by digital microscopy (CoolSnap HQ2; Photometrics, Surrey, BC, Canada) and analyzed by Nikon Elements (Nikon, Melville, NY, USA). Fields of view were chosen to maximize the number of recorded YFP-expression neurons, thus this population is overrepresented in the dissociated neuron functional studies. Changes in [Ca^2+^]_i_ were monitored by sequential dual excitation, 340 and 380 nm (emission 510 nm). The ratio images were acquired every 6 seconds. At the end of each study neurons were exposed to KCl (75 mM, 60 sec) to confirm voltage sensitivity, and ionomycin (5 μM, 60 sec) to obtain a maximal response.

For the analysis of [Ca^2+^]_i_, we used the excitation ratio 340nm/380nm and related all measurements to the peak positive response in each cell. This approach bypasses the conversion of ratiometric responses into absolute [Ca^2+^]_i_ using Tsien parameters, thus we avoided the requirements for calibrating the Fura-2 R_min_, R_max_, K_d_ and β values for each recorded cell. Given that mammalian cells are heterogeneous with respect to their de-esterification of Fura-2AM following uptake, raw ratiometric responses should not be compared quantitatively between individual cells. Thus, as before [33], we have chosen to normalize ratiometric responses at each time point in each cell to its maximum [Ca^2+^]_i_ (evoked by the Ca^2+^ ionophore ionomycin)—data was presented as the percentage change in 340nm/380nm ratio (R): response at time (x) = 100 X (R_x_-R_bl_)/(R_max_-R_bl_), where R_x_ was the 340nm/380nm ratio of the cell at a given time point, R_bl_ was the cell’s mean baseline 340nm/380nm ratio measured over 60s, and R_max_ was the cell’s peak 340nm/380nm ratio. This normalization allows for quantitative comparisons between cells. Only cells that had low [Ca^2+^]_i_ at baseline (R<1.3) and yielded a robust response to the positive control were included in analyses.

A neuron was determined as responding positively to a given ligand if R_ligand_ > R_baseline_ + (2 x SD_baseline_), where R_ligand_ is the mean ratio over the 60s of ligand challenge, R_baseline_ is the mean ratio over the 60s preceding ligand challenge and SD_baseline_ is the standard deviation of the ratio over the 60s preceding ligand challenge.

### Statistical analysis

Data was analyzed using Microsoft Excel and GraphPad software. Where appropriate unpaired Student’s T-test were used. A p value less than 0.05 was taken as significant. All values are mean ± SEM, unless otherwise stated.
